# Active Joint Position Sense in Children With Unilateral Cerebral Palsy

**DOI:** 10.7759/cureus.18075

**Published:** 2021-09-18

**Authors:** Nikolaos Chrysagis, George A Koumantakis, Eirini Grammatopoulou, Emmanouil Skordilis

**Affiliations:** 1 Laboratory of Advanced Physiotherapy (LAdPhys) Physiotherapy Department, School of Health and Care Sciences, University of West Attica (UNIWA), Athens, GRC; 2 School of Physical Education and Sport Science, National and Kapodistrian University of Athens, Athens, GRC

**Keywords:** assessment, spasticity, hemiplegia, position sense, proprioception, cerebral palsy

## Abstract

Objective

The aim of the study was to examine the differences in joint position sense at the elbow joint between 15 children with unilateral cerebral palsy (CP) and 15 typically developing (TD) controls without neurological or other health deficits.

Methodology

Joint position sense, a major proprioceptive component, was evaluated actively using a Kin Com 125 AP isokinetic dynamometer (Chattanooga Group, Chattanooga, TN).

Results

A significant interaction was found (p<0.05) between disability and side, with respect to the active reproduction movement scores. Post-hoc independent t-tests, with Bonferroni adjustments, revealed significant differences for the dominant (t=-3.63, p=0.001) and non-dominant sides respectively (t=-6.19, p=0.000). Repeated measures t-test revealed wider errors with the non-dominant (affected side) in the active reproduction test, compared to the dominant (nonaffected) side for the CP group of children (t=-4.73, p=0.000). A positive correlation was evident between the level of spasticity and joint position sense (Rho=0.71, p=0.003).

Conclusions

Based on our findings, joint position sense is impaired at the elbow joint in children with spastic hemiplegia. The proprioceptive deficit is present at both the affected and unaffected sides and is related to the level of spasticity.

## Introduction

Proprioception refers to the individual's awareness of body parts' position and movement in space, and it is synthesized by two components: kinesthesia and joint position sense. Kinesthesia is the sense of limb movement while joint position sense is the perception of a static limb position [1]. Proprioceptive information is received from the central nervous system by muscle, joint, and tendon receptors, contributing to the design and execution of movements in daily life activities [2,3].

A variety of techniques are available for the assessment of proprioception. According to Han et al. [4], the three most commonly used techniques are as follows: a) detection of passive movement, b) joint position reproduction, and c) active reproduction of movement. Similarly, Hillier et al. [5] proposed three techniques or clusters: a) active or passive joint position reproduction, b) detection of the threshold of passive movement, and c) detection of the direction of movement. Detection of passive movement evaluates the ability of the participant to detect the direction or the movement at various joints of the body at slow speed and with the eyes closed. Joint position reproduction evaluates joint position compared to the ipsilateral or contralateral side, actively or passively, with slow to normal speed, while blindfolded. Finally, functional active reproduction of movement can also be conducted actively with eyes open, but without directly looking at the joint tested (general vision available), and requires the judgment of different movement-displayed distances [6].

The techniques described above, which examine different aspects of proprioception, exhibit certain strengths and limitations according to the experiment employed and the population examined [5]. For example, passive joint reproduction does not require volitional movement while it is conducted passively. On the other hand, assessment of active reproduction requires motor control and represents a more functional use of proprioception. Detection of the direction of movement is easier to administer since it does not depend on memory but upon two choices for the participants involved, e.g., flexion vs. extension [5].

Children with cerebral palsy (CP) may experience proprioception deficits that may disrupt the integration of movement patterns and satisfactory motor control [7]. Further, in the presence of spasticity manifested with inappropriate coactivation of antagonistic muscles, quality of movement and accuracy may be restricted [8]. As a result, manual performance and daily life activities may be adversely affected [3].

Previous research evidence from studies in children with bilateral (tetraplegia/diplegia) or unilateral (hemiplegia) CP has provided conflicting findings with respect to proprioception. The inconsistent findings may be attributed to a number of factors such as the study design, joints examined, and different methods and instruments used [3,9-17]. Wann [11] used the active joint position reproduction method of the contralateral upper limb to compare joint position sense between children with bilateral CP (tetraplegia) and a control group of adults without disabilities. The researcher stated that children with CP exhibited significantly wider errors in the attempt to match a target position with the contralateral upper arm. Similarly, Opila-Lehman et al. [10] reported proprioceptive deficits using passive joint position reproduction in children with tetraplegia. Wingert et al. [12] examined actively joint position sense and detection of passive movement at the elbow and hip joint in children with diplegia. The CP children showed wider errors with both lower limbs and the non-dominant upper limb in joint position sense, compared to controls. Wingert et al. [12] concluded that the kinesthetic deficits were supported only for the upper limbs. More recently, Zarkou et al. [17] have reported significant decrements in the joint position sense of children with diplegia at the ankle joints examined.

On the other hand, Jones [9] stated that the kinesthetic acuity of the upper limbs is similar between children with and without tetraplegia aged 5-11 years. Hall and Gardner [18] used a passive joint reproduction method to examine joint position sense in children with tetraplegia. The researchers found no significant differences in joint position sense between children with tetraplegia and controls [18]. Riquelme et al. [13] found no significant difference between individuals with bilateral CP compared to healthy controls by using a joint reproduction test of the contralateral upper limb. Similarly, Manikowska et al. [14] stated that joint position sense is not affected at the knee joint with exaggerated reflexes in children with bilateral CP. However, a significant difference was present between a control group and children with CP at the knee joint without exaggerated reflexes. De Andrade E Souza Mazuchi et al. [8] examined joint position sense at the elbow joint in children with diplegia. According to the researchers, children with and without CP exhibited similar performance in the active joint reproduction test used [8]. Finally, Zarkou et al. [17] reported no significant deficits in kinesthesia at the ankle joints of children with diplegia.

Only a few studies were retrieved with respect to examining the proprioception in children with hemiplegia, and they usually compared dominant and non-dominant limbs in the absence of a control group or using clinical tests. Specifically, proprioception deficits of 40%, 46%, and 20% were reported in the non-dominant upper limb of children with hemiplegia by Tachdjian and Minear [19], Van Heest et al. [20], and Arnould et al. [3] respectively. Cooper et al. [15] used a clinical test and found kinesthetic deficits on both hands of children with hemiplegia, from four years and four months to 18 years of age compared to age-matched controls. Wingert et al. [12] reported significant differences with regard to joint position sense and kinesthesia at both lower limbs and larger error scores at the upper limbs in children with hemiplegia compared to a control group. Goble et al. [21] and Smorenburg et al. [22] used ipsilateral or contralateral tests to examine the proprioceptive ability at the upper limbs of children with spastic hemiplegia. The researchers found that children with hemiplegia were less accurate in a position-matching task compared to typically developing (TD) children.

Previously, Chrysagis et al. [16] used detection of passive movement and detection of direction of passive movement to examine kinesthesia and passive reproduction of movement at the elbow joints in children with hemiplegia. A significant interaction between participant’s groups and side was reported, indicating kinesthetic deficits on both dominant and non-dominant upper limbs of the children with hemiplegia compared to controls [16]. In the context of the conflicting results mentioned above, the aim of the present study was to shed more light on the field and examine the differences in joint position sense, on both sides, between children with and without hemiplegia using the active reproduction method. Active reproduction is an efficient method for the examination of proprioception, and the active nature of the procedure may increase the ecological validity of the test [4]. Specifically, volitional movement during the test presents a more functional operation of proprioception engaging afferent and efferent mechanisms [5]. Additionally, the present study examined the proprioceptive differences between affected and nonaffected sides of children with hemiplegia and the respective relationship to spasticity and joint position.

## Materials and methods

Participants

A total of 30 children aged 10-15 years participated in the present study. The sample consisted of 15 children (10 boys and five girls) with spastic hemiplegia [Gross Motor Function Classification System (GMFCS) I-II] and 15 age- and gender-matched TD children. Participants' demographic characteristics were published in a previous study [16].

Inclusion criteria for children with spastic hemiplegia were as follows: a) diagnosis of CP (spastic hemiplegia), b) absence of intellectual disability [15], c) ability to follow simple instructions [14], d) spasticity level (1, 1+, and 2) according to the modified Ashworth scale [23], e) ability to grasp and throw a softball, and f) deficits in the full extension of the elbow below 20° [15]. Children without spastic hemiplegia had no history of elbow disorder or pain and no participation in organized sports. The study was approved by the Research Committee of the School of Physical Education and Sport Science at the University of Athens and informed consent was obtained from the participants and their parents.

Measuring tools

The isokinetic dynamometer Kin Com 125 AP Configuration Chattanooga (Chattanooga Group, Chattanooga, TN) [24] was used in the present study. The device constitutes mechanical parts and software that allows active movements in a stable angular velocity commonly used for proprioceptive examination [8]. The dominant upper limb of the TD children was determined by using the Annett Hand Preference Questionnaire (AHPQ) [25]. The questionnaire consists of 12 questions aimed at identifying hand preference in daily activities such as cutting with a scissor.

Procedure

For the purpose of the experiment, the following adaptations were conducted: a seat belt was used for stabilization of the participants in the sitting position and the elbow was aligned with the rotating axis of the dynamometer. The forearm was placed in pronation and a foam material was placed between the wrist and the strap for stabilization of the elbow joint. The foam material was used to minimize the contribution of the skin receptors and secure uniform pressure at the wrist joint. The eyes of the participants were covered during the test [8].

The test was performed on both sides of the participants without vision. The initial position of the elbow was set at 20° due to range of motion deficits at the joint. Participants actively moved the forearm in the two target angles (60° and 100°) with an angular velocity of 25°/second, maintaining the position for five seconds. Then the forearm was returned at the initial position followed by four repetitions at each angle. Two practice trials (for 60° and 100°) were executed for familiarization. The error between the participant’s judgment and the target angle was recorded in a specific form. The overall error score was obtained by the average of the errors at both angles (60° and 100°). Examination of both sides was counterbalanced and the predetermined target angles were tested in a randomized order.

Statistical analysis

The SPSS Statistics version 20 (IBM, Armonk, NY) was used for data analyses. In particular, 2 x 2 factorial analysis of variance (ANOVA) was used to examine the interaction between disability and side, with respect to the active reproduction test. The independent variable disability was constituted based on two levels: a) children with hemiplegia and b) children without hemiplegia, while the independent variable side was constituted based on two levels as well: (a) nonaffected side for the children with hemiplegia and dominant side for the children without hemiplegia and (b) affected side for the children with hemiplegia and non-dominant side for the children without hemiplegia. Differences between children with and without hemiplegia were examined separately for each side with independent t-tests. Further repeated measures t-tests were used to examine differences between the dominant and non-dominant sides of children with CP. Bonferroni adjustments were used in the post-hoc comparisons. The relationship between the level of spasticity and joint position sense was examined with Spearman’s correlation coefficient. The significance level was set initially at the 0.05 level.

## Results

**Figure 1:**
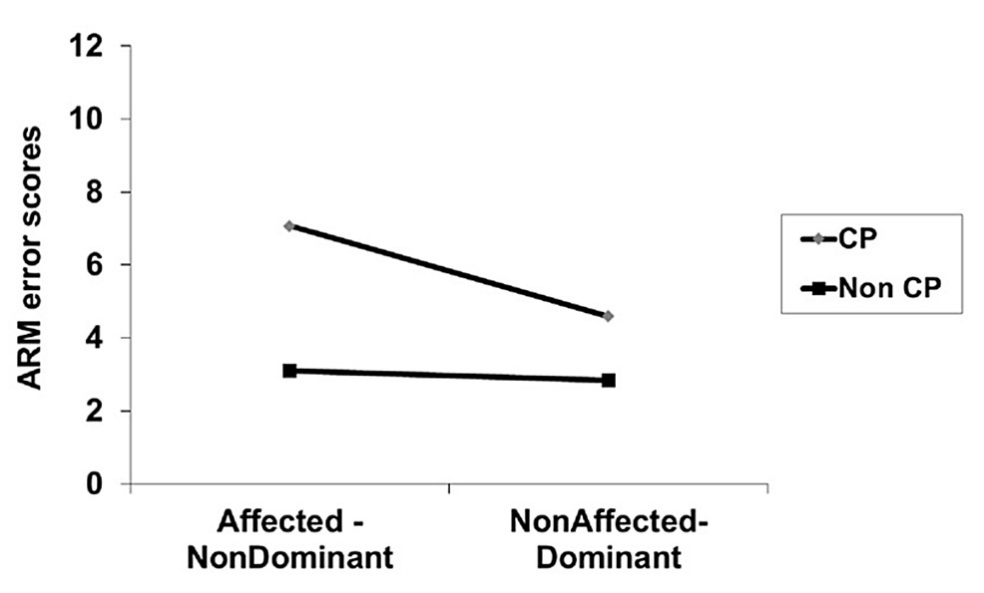
Line graph presenting the interaction between side and disability with respect to the active reproduction method (ARM) of movement error scores (n=30) CP: cerebral palsy

Means of absolute errors for each participant (60° and 100° combined) are presented in Table 1. Further, reliability analysis was conducted separately across groups, sides, and target angles. Intraclass correlation coefficients ranged from 0.57 to 0.72 for the TD group and from 0.57 to 0.65 for the CP group. A significant interaction was found (p<0.05) between disability and side, with respect to the active reproduction movement scores (Table 2, Figure 1).

**Table 1:** Means of the absolute errors for each participant (60° and 100° combined) CP: cerebral palsy

	CP children		Non-CP children	
Participant number	Dominant side	Non-dominant side	Dominant side	Non-dominant side
1	5.38	4.25	1.25	2.50
2	4.50	5.50	3	4.38
3	3.25	6	2.50	2
4	2.75	4	2	3.13
5	6.75	9.63	1.25	1
6	4.25	7.25	2.75	3.88
7	5.75	7.38	1.63	1.13
8	6.88	9.88	4.50	3.87
9	4.88	7.63	4.13	4.62
10	3.63	5.75	3.50	3.50
11	4	5.63	4	3
12	4.25	6.38	3.13	4
13	6.88	10.25	3.38	3.13
14	2	10.63	4.13	5
15	3.75	6	1.63	1.63

**Table 2:** Factorial ANOVA examining the interaction between disability and side for the active reproduction test *Significant at the 0.05 level ANOVA: analysis of variance

Source	F	P	Partial eta squared (η^2^)
Between subjects			
Disability (A)	33.84^*^	0	0.55
Within subjects			
Side (B)	23.84^*^	0	0.46
A x B	15.50^*^	0	0.35

Independent t-tests revealed significant differences for the dominant (t=-3.63, p=0.001) and non-dominant sides respectively (t=-6.19, p=0.000) (Table 3). Significant differences were evident after the Bonferroni adjustment (p<0.025). The non-dominant side of children with CP showed significantly higher errors in the joint position sense compared to their dominant side as revealed by repeated measures t-tests (Table 4). A positive correlation was found between the level of spasticity and error scores in the active reproduction test (Rho=0.708, p=0.003).

**Table 3:** Independent t-tests examining differences in active reproduction test between children with and without CP, separate for each side (df=28) *Significant at the 0.05 level CP: cerebral palsy

Variable	Mean (SD)	Mean diff.	SED	t	P
Dominant		-1.74	0.47	-03.63^*^	0.001
Non-CP	2.85 (1.10)				
CP	4.59 (1.49)				
Non-dominant		-3.95	0.63	-06.19^*^	0
Non-CP	3.11 (1.24)				
CP	7.07 (2.13)				

**Table 4:** Repeated measures t-test, between affected and nonaffected side of CP children (df=14) *Significant at the 0.05 level CP: cerebral palsy

Variable	Mean (SD)	Mean diff.	SE mean	t	P
		-2.48	0.52	-04.73^*^	0
Affected	7.07 (2.13)				
Nonaffected	4.59 (1.49)				

## Discussion

The aim of this study was to examine joint position sense in children with and without hemiplegia by using the active reproduction method. Additionally, we examined differences between the affected and nonaffected sides of children with hemiplegia and the relationship between spasticity and joint position. An interaction effect between disability and side was evident for the active reproduction test. Further, children with hemiplegia exhibited higher errors in both sides compared to the control group. These differences were wider for the non-dominant upper limbs comparisons.

Results of the present study are in agreement with those of Wann [11], Opila-Lehman et al. [10], Wingert et al. [12], and Zarkou et al. [17], who reported joint position sense deficits in the upper and lower limbs of children with bilateral CP. On the contrary, according to Hall and Gardner [18], Riquelme et al. [13], Manikowska et al. [14], and de Andrade E Souza Mazuchi et al. [8], joint position sense, at various joints, is similar between children with and without bilateral CP. However, Hall and Gardner [18] used a passive joint reproduction test while Riquelme et al. [13] examined the ability of the participants to place their upper limbs at the same level. Further, Manikowska et al. [14] and de Andrade E Souza Mazuchi et al. [8] evaluated the participants' ability to move their limbs actively at a predetermined joint angle that was moved passively by the examiner. In contrast with the above, in the present study, the joint position test was conducted in the ipsilateral limb actively throughout the procedure. According to Goble et al. [26], the use of the contralateral limb as a method of position matching in individuals with bilateral CP [11,13] requires larger cooperation between the two hemispheres compared to the ipsilateral technique. Further, the passive movement relies more on cutaneous than muscle spindle sensory information since the muscles are not active. On the contrary, active movement requires mainly muscle spindle input and fusimotor activity as well. As a result, different proprioceptive feedback is delivered to the central nervous system when a limb is passively moved to a target position followed by active reproduction of the target position [4].

Regarding children with hemiplegia, our findings are in agreement with Goble et al. [21] who reported proprioceptive deficits at the affected upper limb of children with hemiplegia. Wingert et al. [12] reported significant deficiencies in joint position sense for both lower limbs and larger error scores for the upper limbs in children with hemiplegia compared to a control group. Further, Smorenburg et al. [22] stated that children with spastic hemiplegia showed lower performance in a contralateral matching test of the upper limbs compared to TD children. However, it should be considered that the above researchers used different examination techniques, procedures, and equipment from those in the present study, such as contralateral matching task [22], self-selected moving speed [12], or improvised constructions [21].

The non-dominant side of children with hemiplegia showed larger errors compared to the dominant side. This finding is in line with Duque et al. [27], Cooper et al. [15], Arnould et al. [3], Wingert et al. [12], and Smorenburg et al. [22], who reported proprioceptive deficits at the nonaffected side of children with hemiplegia, but to a lesser degree than the affected side. On the contrary, Goble et al. [21] found similar performance in an ipsilateral matching test between children with and without hemiplegia.

Joint position deficits found in the present study may arise from primary lesions on the somatosensory cortex in children with hemiplegia. Brain damage disturbs normal sensory processing, affecting motor control development, motor planning, and movement execution [28]. Further, limited previous movement experience of the affected upper limb and poor motor programs may constitute insufficient accuracy on a specific task. Additionally, structural and biochemical changes of the spastic muscles may modify muscle spindles activity, providing inadequate proprioceptive information to the somatosensory cortex [29]. Finally, in the present study, joint position matching was conducted actively. Therefore, co-contraction [2] and inadequate coordination of the muscles may have affected the ability of children with hemiplegia to match the predetermined angles precisely [8].

Spasticity level was correlated with the joint position sense. Similarly, Smorenburg et al. [30] reported a significant correlation between spasticity and active joint position sense of children with hemiplegia. Therefore, the researchers expressed doubts about a clear-cut relationship since some participants with a higher Tardieu score had smaller errors in the proprioceptive task. Spasticity is a velocity-dependent phenomenon and different velocities during the Tardieu or modified Ashworth scale assessment, and/or the active movement during the test, may affect the results. Further, in our study, examination of the joint position sense was conducted in a stable angular velocity (25°/second) while Smorenburg et al. [30] used a self-paced movement during the test.

This study has some limitations, which may have had an effect on the findings; hence, the results should be considered with caution. First, the number of children with and without hemiplegia, although matched for age and gender, was small (n=30), and hence the generalizability of the present findings is limited. Secondly, information on the extent of the brain damage was not available for the CP group; however, the GMFCS level was provided. The extent of brain damage may have been associated with the joint position scores, and future researchers may use that information as a covariate. Third, the attention span, for both CP and non-CP children, was difficult to control. The research team attempted to control that factor by developing a friendly and stimulating environment, minimizing all unnecessary stimuli during the data collection process. Future researchers in the field may consider aiming to overcome the above limitations, by using wider samples of participants as well as imaging methods that may provide useful information regarding the extent and location of the brain damage.

## Conclusions

As per our findings, the proprioception examined with the joint position sense is impaired at the elbow joint in children with spastic hemiplegia. The deficit, present at both sides, is more evident in the affected limb. Further, joint position sense is related to the level of spasticity measured with the modified Ashworth scale. Clinicians need to consider the above findings and plan their rehabilitative work by assessing both sides in hemiplegic children. In turn, they may need to provide the necessary stimuli, during the rehabilitation program, to enhance the perception of static and dynamic limb positions on both sides in CP hemiplegic children.
